# Selection response and estimation of the genetic parameters for multidimensional measured breast meat yield related traits in a long-term breeding Pekin duck line

**DOI:** 10.5713/ajas.17.0837

**Published:** 2018-04-12

**Authors:** Yaxi Xu, Jian Hu, Yunsheng Zhang, Zhanbao Guo, Wei Huang, Ming Xie, Hehe Liu, Chuzhao Lei, Shuisheng Hou, Xiaolin Liu, Zhengkui Zhou

**Affiliations:** 1 College of Animal Science and Technology, Northwest A&F University, Yangling, Shaanxi 712100, China; 2 Institute of Animal Sciences, Chinese Academy of Agricultural Sciences, Beijing 100193, China

**Keywords:** Pekin Duck, Breast Meat, Ultrasound Scanning, Genetic Parameter, Breeding Value

## Abstract

**Objective:**

This study was conducted to estimate the genetic parameters and breeding values of breast meat related traits of Pekin ducks. Selection response was also determined by using ultrasound breast muscle thickness (BMT) measurements in combination with bosom breadth (BB) and keel length (KL) values.

**Methods:**

The traits analyzed were breast meat weight (BMW), body weight (BW), breast meat percentage (BMP) and the three parameters of breast meat (BB, KL, and BMT). These measurements were derived from studying 15,781 Pekin ducks selected from 10 generations based on breast meat weight. Genetic parameters and breeding value were estimated for the analysis of the breeding process.

**Results:**

Estimated heritability of BMW and BMP were moderate (0.23 and 0.16, respectively), and heritability of BW was high (0.48). Other traits such as BB, KL, and BMT indicated moderate heritability ranging between 0.11 and 0.28. Significant phenotypic correlations of BMW with BW and BMP were discovered (p<0.05), and genetic correlations of BMW with BW and BMP were positive and high (0.83 and 0.66, respectively). It was noted that BMW had positive correlations with all the other traits. Generational average estimated breeding values of all traits increased substantially over the course of selection, which demonstrated that the ducks responded efficiently to increased breast meat yield after 10 generations of breeding.

**Conclusion:**

The results indicated that duck BMW had the potential to be increased through genetic selection with positive effects on BW and BMP. The ultrasound BMT, in combination with the measurement of BB and KL, is shown to be essential and effective in the process of high breast meat yield duck breeding.

## INTRODUCTION

Duck meat consumption, on a global scale, has continued to rise over the last few decades. Duck meat has been considered a functional food due to its composition of fatty acids. The composition includes a high proportion of polyunsaturated fatty acids and a favorable ratio of omega 6 to omega 3-fatty acids [1–3]. The increasing demand for poultry meat has forced breeders to improve the growth rate and the meat yield of ducks, with the carcass meatiness being reflected primarily in the content of breast and leg meat. Representing the largest portion of meat in ducks, breast muscle weight has always been considered an important trait in duck breeding processes.

Direct measurement of breast meat weight was initially difficult and could only be achieved after the slaughter. Thus, progeny testing was the only way to selectively breed for higher breast meat yield. This had shortcomings in terms of high cost, time and operational complexity. Fortunately, breeders found that breast meat yield was positively correlated with breast muscle thickness (BMT). Therefore, selection for higher breast meat yield could be achieved by measuring the thickness of breast muscle [4]. The thickness of the breast muscle was originally determined by a needle probe which brought great harm to live birds. Ultrasound has since replaced this method as it has shown to improve the convenience and accuracy of the body composition prediction [5–10].

Besides the BMT, breast meat yield is determined by keel length (KL), and breast breadth (BB). A comprehensive selection index combining the three factors might be more efficient in selection for higher breast meat yield, and should be based on a good understanding of genetic parameters of breast meat related traits. Recently, the genetic parameters of growth traits have been studied in poultry, especially in chicken [11–13]. In ducks, previous studies have concentrated on estimating genetic parameters for body weight (BW), carcass composition, and egg production traits [14–16]. Systematic studies on genetic parameters of breast meat related traits in a long-term breeding Pekin duck populations are lacking.

In the Pekin duck breeding process employed for this study, breast muscle weight has been selected for higher breast meat yield over 10 generations. Compared with previously described breeding methods for high breast meat yield in ducks, the methods incorporated in this study additionally combined BB and KL. The BMT was measured through ultrasound scanning to estimate breast meat yield. The objectives of this study were not only to provide estimated genetic parameters for breast meat related traits, but also to estimate the selection response of traits in the long-term Pekin duck breeding population based on data from ducks over a period of 10 generations. Outcomes were expected to provide reference data for methods used in previous breeding programs and some guidance for future breeding strategies.

## MATERIALS AND METHODS

### Animals

Z2 Pekin duck line has been selected for high breast meat yield over 10 generations in the Pekin duck breeding farm at the Institute of Animal Science, Chinese Academy of Agricultural Sciences. The initial population was the local Pekin duck population in Beijing. The line was closed after formation in the first generation of selection. The ducks produced one generation a year. Based on breast meat volume (BMV) measured at 6 week of age, about 320 top female ducks and 80 top male ducks in each generation were selected from about 1,600 ducks (about 800 males and 800 females) as parents of the next generation. A ratio of 1:4 of males to females was used in each sire family. Besides that, inbreeding was strictly avoided through calculating the inbreeding coefficient of population in each generation. The 320 female ducks and 80 male ducks were placed in a mating room at 20 week of age. In one sire family, the 4 female ducks were separated in four cages, while the male duck moved one cage a day, 4 days a round. After two weeks, the eggs labeled with the cage number were collected every day for hatching. The hatching eggs were stored at 4°C and then all eggs were sent into the incubator for hatching at one time. At 20th day of hatching, the day before the first duckling hatched, the eggs labeled with same cage number were put into one labeled plastic mesh pocket for hatching. Therefore, every hatched duckling had clear pedigree information.

All the ducks were kept in plastic-wire-floor pens in an environmentally controlled duck shed from 1 to 21 d of age, in the duck house with litter floors from 22 to 42 d of age. The temperature in the duck shed was set at 28°C from 1 to 3 d of age, at 26°C from 4 to 7 d of age, at 25°C from 8 to 21 d of age, then kept at room temperature in the later 2 weeks. All ducks had free access to pelleted feed and were provided with continuous drinking water and lighting. Ducks in each generation were fed *ad libitum* with the same commercial diets.

Records on 15,781 individual Z2 Pekin ducks from 2005 to 2015 were used for this analysis.

### Collection of phenotypic traits

The duck traits in the populations were measured at 6 week of age corresponding to their respective growth features. Ducks were weighed after 12 hours off-feed. All traits were measured *in vivo*. Using a Vernier caliper, the BB and KL were measured, while BMT was measured by the AQUILA VET (Esaote Europe B.V., Maastricht, Netherlands) ultrasound system, a kind of B ultrasound scanning technology. BMW was estimated according to the BMV derived from BB, KL, and BMT values. The equation for determining BMV was:

$$\begin{array}{l} \text{BMV}=\text{BB}\times \text{KL}\times \text{BMT}\\ \text{BMW}=0.6228\times (\text{BB}\times \text{KL}\times \text{BMT})+17.042 \end{array}$$

**Figure 1:**
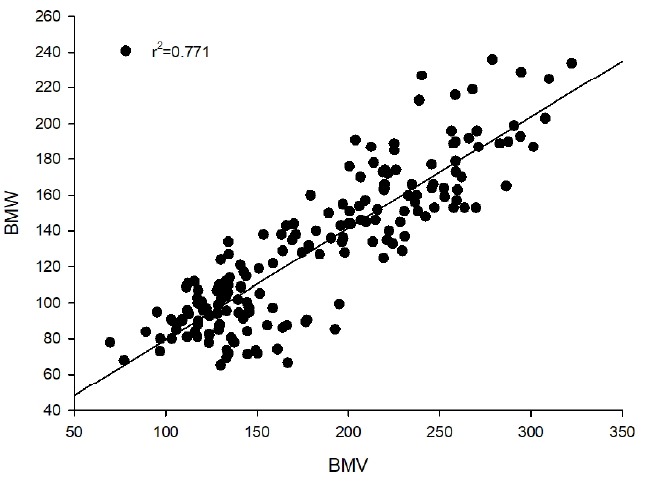
Breast meat weight (BMW) vs breast meat volume (BMV) and the regression line from BMV to BMW. r^2^ = 0.771.

This method was based on the high correlation between real BMW and BMV obtained from slaughter experiments over 10 years. Real BMW were measured after slaughter. BMW was obtained from the linear regressive equation model describing the relationship between BMV and BMW. Figure 1 shows the regression analysis result from BMV to BMW. The coefficient of determination was found to be 0.771.

After all traits collected, individuals with traits data beyond three standard deviations from the estimated sample mean in each generation, considered as outliers, were removed from the data set.

Each fertile egg had clear records about its parents. Therefore, we had clear pedigree of the duck line. All pedigree information was taken into account during the heritability prediction and breeding value estimation.

### Software and model

In this study, Multiple Trait Derivative-Free Restricted Maximum Likelihood (MTDFREML), a set of programs used to estimate (co)variance components using animal models and derivative-free REML, was utilized to estimate the genetic parameters of traits in this study [17]. The model used was a bivariate animal model. The model used was

$$\left[\begin{array}{c} y_{1}\\ y_{2} \end{array}\right]=\left[\begin{array}{cc} X_{1} & 0\\ 0 & X_{2} \end{array}\right]\;\left[\begin{array}{c} b_{1}\\ b_{2} \end{array}\right]+\left[\begin{array}{cc} Z_{1} & 0\\ 0 & Z_{2} \end{array}\right]\;\left[\begin{array}{c} a_{1}\\ a_{2} \end{array}\right]+\left[\begin{array}{c} e_{1}\\ e_{2} \end{array}\right]$$

Where, *y*_1_, *y*_2_ were vectors of the observations for trait 1 and 2, respectively; *X*_1_, *X*_2_ were incidence matrixes of fixed effects for trait 1 and 2, respectively; *b*_1_, *b*_2_ were vectors of fixed effects associated with records in *y* by *X* for trait 1 and 2, respectively; *Z*_1_, *Z*_2_ were incidence matrixes of animal genetic effects for trait 1 and 2, respectively; *a*_1_, *a*_2_ were vectors of random effects associated with records in *y* by *Z* for trait 1 and 2, respectively; *e*_1_, *e*_2_ were vectors of random error for trait 1 and 2, respectively.

$$\begin{array}{l} \text{E }({\text{y}}_{1})={\text{X}}_{1}{\text{b}}_{1},\;\;\;\text{E }({\text{y}}_{2})={\text{X}}_{2}{\text{b}}_{2},\\ \text{V }\left[\begin{array}{c} a_{1}\\ a_{2}\\ e_{1}\\ e_{2} \end{array}\right]=\left[\begin{array}{cccc} A\sigma_{a_{1}}^{2} & A\sigma_{a_{1}a_{2}} & 0 & 0\\ A\sigma_{a_{1}a_{2}} & A\sigma_{a_{2}}^{2} & 0 & 0\\ 0 & 0 & I\sigma_{e_{1}}^{2} & I\sigma_{e_{1}e_{2}}\\ 0 & 0 & I\sigma_{e_{1}e_{2}} & I\sigma_{e_{1}}^{2} \end{array}\right] \end{array}$$

Where *A* is the additive relationship matrix constructed from pedigree data of the 15,781 ducks, I is an identity matrix, $\sigma_{a_{1}}^{2}$ and $\sigma_{a_{2}}^{2}$ were direct additive genetic variances, $\sigma_{e_{1}}^{2}$ and $\sigma_{e_{1}}^{2}$ were the residual variances for the traits, respectively; *σ_a_*__1__*_a_*__2__ was the direct genetic correlation between the traits, and *σ_e_*__1__*_e_*__2__ was the residual covariance of the traits.

Heritability and genetic correlations were predicted using the following equations:

$$\begin{array}{l} {\text{h}}^{2}=\frac{\sigma_{a}^{2}}{\left(\sigma_{a}^{2}+\sigma_{\text{e}}^{2}\right)}\\ {\text{r}}_{\text{a}}=\frac{\sigma_{a_{1}a_{2}}}{\sigma_{a_{1}}\sigma_{a_{2}}} \end{array}$$

The fixed effects included sex and generation. There were 2 sexes and 10 generations in this line.

## RESULTS

### Phenotypic data

The descriptive statistics for studied traits (including number of observations, mean, standard deviation, coefficient of variation, and minimum and maximum value) are presented in Table 1. The coefficients of variation (CV) of measured traits ranged from 4.67% to 18.82%. The highest CV was observed at the target trait—BMW (18.82%). The CV was second highest in BMP (12.53%), and third highest in BW (12.14%).

### Genetic parameters

Heritability, genetic correlations, and phenotypic correlations of all traits are shown in Table 2. The heritability estimates for breast meat related traits ranged from 0.12 to 0.28. Among these traits, BMW, BB, and KL had similar heritability (0.26, 0.28, and 0.26, respectively). BMP had moderate heritability with 0.16, and the heritability estimate for BMT was the lowest at only 0.12. Besides the breast meat related traits, BW had the highest heritability with 0.48. The three parameters of chest shape showed low heritability with 0.11, 0.09, and 0.08, respectively.

In this study, BMW showed high genetic and phenotypic association with BW (0.83 and 0.80) and BMP (0.66 and 0.85). BMW had high genetic correlation with BB, KL, and BMT (0.89, 0.88, and 0.86, respectively). In contrast with breast meat related traits, BW showed low genetic correlation (0.08) with BMP.

### Generational average estimated breeding values

**Figure 2:**
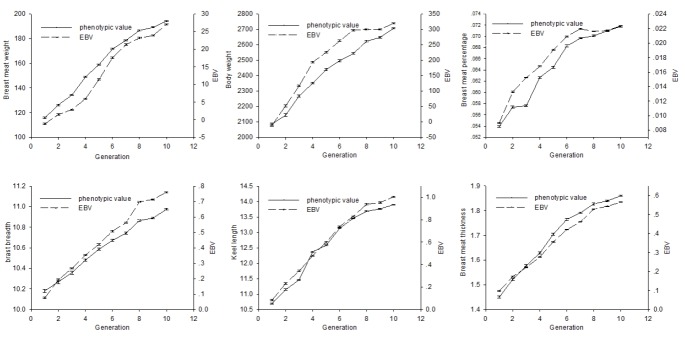
Generational average phenotypic values and estimated breeding values (EBVs), reflecting genetic trends for breast meat related traits. Vertical lines denote standard errors.

Generational average estimated breeding values (EBVs) of BW and breast meat related traits are shown with broken lines and the generational average phenotypic values of traits are shown with the full lines in Figure 2. Vertical lines denoted standard errors, which were very small. Both the phenotypic values and generational average EBVs of BMW, BW, BMP, BB, KL, BMT continued to increase across the 10 generations. But from the overall trend, the speed of the increase of all traits slowed down at the later period.

## DISCUSSION

### Heritability estimates

As shown in Table 2, the heritability estimate for BMW (0.26) was relatively high for a moderately heritable trait; therefore, genetic improvement of breast meat weight could be obtained from direct selection. The heritability of BMW estimated in this study was similar to the results described by Gaya et al [18], but was much smaller than that estimated in chicken by Le Bihan-Duval et al [19], and in Pekin duck by Xu et al [16]. High heritability was observed for BW (0.48), consistent with the results estimated by Zhang et al [20], Xu et al [16], Wolc et al [21], and Grupioni et al [22] in chicken, Pekin duck, layer chicken, and broiler populations, respectively [16,20–22]. Heritability estimates for BW shown by Gaya et al [18], Barbato [11], and Quinton et al [23] ranged from 0.18 to 0.24, much smaller than 0.48 indicated in this study. The heritability estimates for BMT and BMP were 0.12 and 0.16, respectively, which were much lower than the results (0.45 and 0.47, respectively) shown by Xu et al [16]. Complex reasons might account for the differences between the heritability estimates. Mignon-Grasteu et al [24] showed that, in chicken, the heritability difference of BW was caused by age and sex. Le Bihan-Duval et al [19] also observed variation in the heritability of BW and BMW between different ages and sexes. Barbieri et al [13] showed that the heritability estimates for BW at 7 ages from day 0 to day 42 varied in commercial quail populations. Similar results were observed by others [11,23]. According to previous studies, heritability estimates for one trait could varied for different ages, populations, feeding environments and so on.

This indicated that BB and KL could be improved under the same selection pressure. It could therefore be more advantageous to take BB and KL into consideration in the selection process for increased breast meat yield.

### Genetic correlation estimates

BMW had high genetic and phenotypic association with BW (0.83 and 0.80) and BMP (0.66 and 0.85), which indicated that the selection for BMW could also result in the improvements of BW and BMP. This result corresponded with a previous study [16]. In contrast with breast meat related traits, BW showed low genetic and phenotypic association with BMP (0.08 and 0.37). Although not meaningfully contributing to BMP, it could be concluded that selection for BW might produce improved BMW. BB, KL, and BMT all indicated strong genetic and phenotypic correlation with BMW, suggesting that selective breeding for these three parameters all contribute to the improvement of BMW. According to the genetic and phenotypic correlation between traits as presented in this study, higher breast meat yield might be achieved by combining BB, KL, and BMT selection traits.

### Genetic trends during the breeding process

Individual breeding values depict direct genetic change. Cloete et al [25] predicted breeding values for live weight and reproduction in ostrich populations to reveal the direct responses to selection. Collectively, the estimated breeding value could reflect the selection response throughout the breeding process.

The constant increase in the average EBV observed during the breeding process across generations indicates the genetic improvement in BMW. This was consistent with the moderate heritability (0.26) of BMW. The generational average EBV of BMW increased from −1.18 g in the first generation to 30.22 g in the eleventh generation. Remarkable improvements in BW and BMP were also obtained during the breeding process. Spanning 10 generations, the generational average EBV of BW and BMP increased to 349.45 g and 1.41%, respectively. This phenomenon could be explained by the high genetic and phenotypic correlation between BW and BMW, and BMP and BMW (0.83 and 0.80, 0.66 and 0.85, respectively). BB, KL, and BMT served as three important parameters in estimating breast meat weight, and together reflected the shape of breast meat. During the breeding process for higher breast meat yield, the generational average EBV of BB, KL, and BMT increased by 0.70 cm, 0.90 cm, and 0.50 cm, respectively. This phenomenon indicated that all three parameters had been positively selected for during the breeding process. Collectively, the selection program on BMW used here appears to be effective for breeding Pekin ducks with high breast meat yield.

Besides that, the increasing trends of all traits slowed down at the later period, which meant that the selection became increasingly difficult at the later period of breeding process. This phenomenon was consistent with the previous studies and could be explained by the decreasing genetic variation of the population along with the selection [21,25,26]. The ratios from KL to BB, from BMT to KL, from BMT to BB all increased during the breeding process. The increasing trend of the ratios indicated that BMT increased more rapidly than KL, and that KL increased more rapidly than BB during the breeding process. This trend implies that the breast meat became thicker and longer during the breeding process. BB was more difficult to improve than KL and BMT.

In conclusion, BMW could be modified by selection, and selecting for BMW could simultaneously improve BW and BMP. BB, KL, and BMT all influence breast meat yield, while selection programs combining these three factors are essential for higher breast meat yield. Additionally, in the selection of traits for increased breast meat yield in this study, the combined use of the ultrasound scanning methods with BB and KL measurements proved to be effective. Based on the previous breeding processes, this study provides some references and guidance for future breeding programs.

## Figures and Tables

**Table 1:** Statistical summary of performance of breast meat related traits

Trait	N	Mean	SD	CV (%)	Min	Max
BMW (g)	13,838	170.7	32.1	18.82	60.5	282.6
BB (cm)	13,847	10.71	0.50	4.67	7.48	13.10
KL (cm)	13,847	13.02	1.18	9.07	8.41	17.57
BMT (cm)	13,840	1.75	0.20	11.21	0.92	2.58
BW (g)	15,516	2496	303	12.14	1124	3888
BMP (%)	13,573	6.69	0.84	12.53	2.87	11.04

SD, standard deviation; CV, coefficients of variation; BMW, breast meat weight; BB, bosom breadth; KL, keel length; BMT, breast muscle thickness; BW, body weight at 42 days; BMP, breast meat percentage.

**Table 2:** Estimates of heritabilities1) (on the diagonal), genetic correlations2) (below the diagonal) and phenotypic correlations (above the diagonal) obtained for breast meat related traits

Traits	BMW	BB	KL	BWT	BW	BMP
BMW	0.26	0.77	0.90	0.90	0.80	0.85
BB	0.89	0.28	0.70	0.51	0.73	0.56
KL	0.88	0.84	0.26	0.66	0.77	0.73
BMT	0.86	0.63	0.58	0.12	0.63	0.84
BW	0.83	0.82	0.76	0.72	0.48	0.37
BMP	0.66	0.52	0.54	0.63	0.08	0.16

BMW, breast meat weight; BB, bosom breadth; KL, keel length; BMT, breast muscle thickness; BW, body weight; BMP, breast meat percentage.

1) Standard errors of the estimated heritability ranged from 0.012 to 0.021.

2) Standard errors of the estimated genetic correlations ranged from 0.000 to 0.022.

## References

[1] Barroeta AC (2007). Nutritive value of poultry meat: relationship between vitamin e and pufa. Worlds Poult Sci J.

[2] Givens I (2009). Animal nutrition and lipids in animal products and their contribution to human intake and health. Nutrients.

[3] Petracci M, Cavani C (2012). Muscle growth and poultry meat quality issues. Nutrients.

[4] Pingel H (2011). Results of selection for breast muscle percentage and feed conversion ratio in pekin ducks. Biotechnol Anim Husb.

[5] Wilson DE (1992). Application of ultrasound for genetic improvement. J Anim Sci.

[6] Farhat A, Chavez ER (2001). Metabolic studies on lean and fat pekin ducks selected for breast muscle thickness measured by ultrasound scanning. Poult Sci.

[7] Hou SS, Huang W, Fan H (2004). Study on the Relationship between the breast muscle thickness and the Carcass Performance of Pekin duck. Chinese J Anim Vet Sci.

[8] Farhat A (2009). Carcass characteristics of pekin ducks selected for greater breast muscle thickness using ultrasound scanning in response to dietary protein. Res J Agric Biol Sci.

[9] Kleczek K, Wawro K, Wilkiewicz-Wawro E (2009). Relationships between breast muscle thickness measured by ultrasonography and meatiness and fatness in broiler chickens. Arch Tierz.

[10] Gaya LDG (2013). Genetic variability in ultrasound records of breast muscle in a broiler breeding program. Nat Sci.

[11] Barbato GF (1991). Genetic architecture of growth curve parameters in chickens. Theor Appl Genet.

[12] Bennewitz J, Morgades O, Preisinger R (2007). Variance component and breeding value estimation for reproductive traits in laying hens using a bayesian threshold model. Poult Sci.

[13] Barbieri A, Ono RK, Cursino LL (2015). Genetic parameters for body weight in meat quail. Poult Sci.

[14] Cheng YS, Rouvier R, Poivey JP (1995). Genetic-parameters of body-weight, egg-production and shell quality traits in the brown tsaiya laying duck. Genet Sel Evol.

[15] Hu YH, Poivey JP, Rouvier R (1999). Heritabilities and genetic correlations of body weights and feather length in growing muscovy selected in taiwan. Br Poult Sci.

[16] Xu TS, Liu XL, Huang W (2011). Estimates of genetic parameters for body weight and carcass composition in pekin ducks. J Anim Vet Adv.

[17] Groeneveld E (1990). PEST users’ manual.

[18] Gaya LG, Ferraz JBS, Rezende FM (2006). Heritability and genetic correlation estimates for performance and carcass and body composition traits in a male broiler line. Poult Sci.

[19] Le Bihan-Duval E, Mignon-Grasteau S, Millet N (1998). Genetic analysis of a selection experiment on increased body weight and breast muscle weight as well as on limited abdominal fat weight. Br Poult Sci.

[20] Zhang W, Aggrey SL, Pesti GM (2003). Genetics of phytate phosphorus bioavailability: Heritability and genetic correlations with growth and feed utilization traits in a randombred chicken population. Poult Sci.

[21] Wolc A, Stricker C, Arango J (2011). Breeding value prediction for production traits in layer chickens using pedigree or genomic relationships in a reduced animal model. Genet Sel Evol.

[22] Grupioni NV, Cruz VAR, Stafuzza NB (2015). Phenotypic, genetic and environmental parameters for traits related to femur bone integrity and body weight at 42 days of age in a broiler population. Poult Sci.

[23] Quinton CD, Wood BJ, Miller SP (2011). Genetic analysis of survival and fitness in turkeys with multiple-trait animal models. Poult Sci.

[24] Mignon-Grasteu S, Beaumont C, Le Bihan-Duval E (1999). Genetic parameters of growth curve parameters in male and female chickens. Br Poult Sci.

[25] Cloete SWP, Brand Z, Bunter KL (2008). Direct responses in breeding values to selection of ostriches for liveweight and reproduction. Aust J Exp Agric.

[26] Biscarini F, Bovenhuis H, Ellen ED (2010). Estimation of heritability and breeding values for early egg production in laying hens from pooled data. Poult Sci.

